# Risk factors for cervical lymph node metastasis in oropharyngeal cancer and its impact on prognosis

**DOI:** 10.1016/j.bjorl.2024.101520

**Published:** 2024-11-05

**Authors:** Li Zhang, Zhilin Li, Jing Wang, Chen Wang, Shuxin Wen

**Affiliations:** a Shanxi Medical University, Taiyuan, China; b Shanxi Province Cancer Hospital, Department of Head and Neck Surgery, Taiyuan, China; c Shanxi Hospital Affiliated to Cancer Hospital, Chinese Academy of Medical Sciences, Department of Head and Neck Surgery, Taiyuan, China; d Cancer Hospital Affiliated to Shanxi Medical University, Department of Head and Neck Surgery, Taiyuan, China; e Third Hospital of Shanxi Medical University, Department of Otolaryngology Head and Neck Surgery, Taiyuan, China; f Shanxi Bethune Hospital, Department of Otolaryngology Head and Neck Surgery, Taiyuan, Shanxi, China; g Shanxi Province Cancer Hospital, Department of Pathology, Taiyuan, China

**Keywords:** Oropharyngeal squamous cell carcinoma, Cervical lymph node metastasis, P16 protein

## Abstract

**Objective:**

To understand the characteristics of cervical lymph node metastasis in Oropharyngeal Squamous Cell Carcinoma (OPSCC) patients, explore the risk factors for lymph node metastasis, and its impact on prognosis.

**Methods:**

Retrospective cohort study. Statistical analysis of the relationship between various clinicopathological factors and lymph node involvement, as well as the independent risk factors affecting lymph node metastasis and their impact on prognosis.

**Results:**

The cervical lymph node metastasis rate in this group was 89.2%, with the palatine tonsils and base of the tongue more prone to cervical lymph node metastasis (*p* = 0.007); lower pathological differentiation of OPSCC was more likely to develop cervical lymph node metastasis (*p* = 0.017). The P16 protein positive rate in this group was 58.8%. P16 protein expression did not affect the incidence rate of cervical lymph node metastasis in OPSCC (*p* = 1.000) but influenced the risk of multiple lymph node metastases (*p* = 0.011) and was a risk factor affecting the prognosis of OPSCC (*p* = 0.003). However, the lymph node metastasis status did not affect the survival time of OPSCC patients.

**Conclusion:**

In the Shanxi region of China, HPV-related OPSCC accounts for a higher proportion; OPSCC has a high rate of cervical lymph node metastasis, but lymph node metastasis does not significantly increase the mortality risk of OPSCC patients, which may be related to the unique characteristics of HPV-related OPSCC.

**Level of Evidence Ⅳ:**

Retrospective cohort study.

## Highlights

• The proportion of HPV-positive OPSCC in the Shanxi region has reached 58.8%.
• The status of lymph node metastasis has no significant impact on prognosis.
• P16 status does not affect the incidence of cervical lymph node metastasis.
• P16 positive reduces the risk of multiple lymph node metastases.

## Introduction

Oropharyngeal Squamous Cell Carcinoma (OPSCC) is one of the most common malignant tumors in the head and neck region, with both incidence and mortality rates among the highest for head and neck cancers. In 2020, there were approximately over 100,000 new cases of OPSCC worldwide [1]. Over the past 30 years, the incidence of oropharyngeal cancer has been rising, with studies in Europe and America linking the increase to Human Papillomavirus (HPV) infection [2]. Further research has shown that HPV-Positive OPSCC (HPV-OPSCC) has distinct biological characteristics and prognosis compared to OPSCC caused by traditional factors like smoking and alcohol [3]. This led to the American Joint Committee on Cancer (AJCC) creating separate staging for HPV-positive OPSCC in the 8th edition of the TNM staging system for Head and Neck Squamous Cell Carcinoma (HNSCC), with significant differences in staging of the primary Tumor site (T) and cervical lymph Node involvement (N) for HPV-OPSCC [4]. Particularly noteworthy are the changes in N staging for HPV-OPSCC, where the size, number, and location of metastatic lymph nodes show distinct variations. Cervical lymph node metastasis is one of the most critical prognostic factors in HNSCC, but in the 8th AJCC, TNM staging for HPV-OPSCC suggests that lymph node metastasis may not significantly affect prognosis in HPV-OPSCC as it does in other HNSCCs.

This study retrospectively analyzes the characteristics and risk factors of cervical lymph node metastasis in OPSCC patients treated in two tertiary hospitals in Shanxi Province in recent years. By examining the expression of P16 and Ki-67 proteins and integrating the clinical TNM staging for HPV-OPSCC from the 8th AJCC, the research aims to understand the features of lymph node metastasis in Shanxi's OPSCC patients and its correlation with prognosis. Additionally, it analyzes the compatibility of the 8th AJCC oropharyngeal cancer TNM staging, aiming to provide a basis for better personalized treatment for OPSCC patients.

## Methods

### General information

This study selected 102 hospitalized patients diagnosed with squamous cell carcinoma of the oropharynx through pathological confirmation at the Shanxi Cancer Hospital and Shanxi Bethune Hospital from January 2017 to August 2023 as the research subjects. The ethical committees of Shanxi Cancer Hospital and Shanxi Bethune Hospital have approved this study.

Inclusion criteria: (A) Pathologically confirmed primary OPSCC with complete medical records; (B) Underwent a complete diagnostic and treatment process, with pre-treatment enhanced CT/MRI of the neck and chest, neck ultrasound, or PET/CT examinations, evaluated by two experienced radiologists for cervical lymph node status (either no lymph nodes or suspicious lymph nodes on the same side as the lesion without extracapsular invasion); (C) Possession of biopsy or postoperative histopathological specimens; (D) Patients and their families were fully informed about the comprehensive treatment and postoperative follow-up of OPSCC and signed a treatment consent form.

### P16 and Ki-67 immunohistochemical testing

Pathological wax blocks are cut into 4 μm thick sections and baked at 72 °C for 45 min on a slide spreader. The deparaffinization, hydration, antigen retrieval, and application of primary and secondary antibodies are completed using an automatic immunohistochemistry machine, proceeding until DAB coloring is finished. The slides are then removed from the machine, rinsed with tap water, and stained in hematoxylin for 1 min. After another rinse with tap water and differentiation in the differentiator for 1–2 seconds, the slides are rinsed again, set still for bluing for 10–15 min, and then placed in an immunohistochemistry restainer for dehydration and clearing. The dehydrated and cleared slides are then placed in an automatic sealing machine for mounting. The slides are read under a microscope.

Results interpretation: P16 protein is considered positive when over 70% of the tumor cell nuclei and cytoplasm exhibit strong diffuse staining. The Ki-67 positivity rate is determined by the percentage of tumor cells with brownish-yellow or brown granules in the nucleus indicating positive cells.

### Treatment

According to the NCCN guidelines, surgery or radiation alone may be considered for T1-2N0 cases, surgery, radiation, or concurrent chemoradiotherapy for T1-2N1 cases, while locally advanced T3-4a or N2-3 cases may consider surgery, concurrent chemoradiotherapy, or sequential chemoradiotherapy following induction chemotherapy.

### Follow-up

The first follow-up is conducted one month after treatment completion, followed by quarterly visits in the first year, biannual visits within five years, and annual visits thereafter.

### Statistical methods

The endpoint of this study is Overall Survival (OS), defined as the time from the initial diagnosis of the disease to death from any cause or the last follow-up. Patients were grouped based on the lymph node status assessed by imaging at diagnosis (lymph node positive or negative, single or multiple, and metastatic lymph node diameter ≤3 cm or >3 cm), with the population divided into two groups by the median age of 58 years and primary tumor anatomical subsites divided by the degree of lymph follicle enrichment. SPSS 25.0 software package was used for statistical analysis, employing univariate and multivariate Logistic regression to examine the relationship between various clinicopathological factors and lymph node status, as well as independent risk factors affecting lymph node metastasis status. Kaplan–Meier method was used for survival analysis of OPSCC patients, with *p* < 0.05 considered statistically significant.

## Results

### Clinicopathological characteristics of OPSCC patients

The clinicopathological characteristics of the 102 patients are presented in Table 1.

**Table 1:** OPSCC patient lymph node metastasis characteristics risk factor analysis. Grouping of 102 patients based on lymph node status assessed through imaging at diagnosis (lymph node positive or negative, single or multiple, and metastatic lymph node diameter ≤3 cm or >3 cm). The population is divided into two groups by the median age of 58 years and the primary tumor anatomical subsites divided by the degree of lymph follicle enrichment. Univariate logistic regression analysis was conducted to identify risk factors affecting lymph node metastasis status in OPSCC patients.

Clinicopathological		Total = 102	Lymph node metastasis		Multiple lymph node metastasis		Lymph node metastasis greatest diameter > 3 cm	
Parameters		n (%)	Positive (%)	*p*	Positive (%)	*p*	Positive (%)	*p*
Gender	Male	88 (86.3)	79 (77.5)	0.651	16 (15.7)	0.707	13 (12.7)	**0.124**
	Female	14 (13.7)	12 (11.8)		3 (2.9)		3 (2.9)	
Age (years)	≤58	53 (52.0)	47 (46.1)	0.856	11 (10.8)	0.541	8 (7.8)	0.331
	>58	49 (48.0)	44 (43.1)		8 (7.8)		8 (7.8)	
Smoking	No	42 (41.2)	36 (35.3)		8 (7.8)		6 (5.9)	
	PY <10	11 (10.8)	11 (10.8)	0.993	3(2.9)	0.73	1 (1.0)	0.994
	PY ≥10	49 (48.0)	44 (43.1)	0.553	8 (7.8)	0.654	9 (8.8)	0.431
Alcohol	No	64 (62.7)	55 (53.9)	0.183	11 (10.8)	0.799	9 (8.8)	0.396
	Yes	38 (37.3)	36 (35.3)		8 (7.8)		7 (6.9)	
Site	Tonsil	75 (73.5)	71 (69.6)	***0.007***	13 (12.7)	0.261	10 (9.8)	0.135
	Base of tongue							
	Soft palate	27 (26.5)	20 (19.6)		6 (5.9)		6 (5.9)	
	posterior pharyngeal wall							
cT stage	T1/T2	83 (81.4)	73 (71.6)	0.404	15 (14.7)	0.876	15 (14.7)	0.992
	T3/T4	19 (18.6)	18 (17.6)		4 (3.9)		1 (1.0)	
Distant metastasis	No	92 (90.2)	83 (81.4)	0.334	14 (13.7)	***0.007***	13 (12.7)	0.143
	Yes	10 (9.8)	8 (7.8)		5 (4.9)		3 (2.9)	
Tumor Grade	Highly	10 (9.8)	7 (6.9)		2 (2.0)		1 (1.0)	
	Moderately	39 (38.2)	33 (32.4)	0.296	8 (7.8)	0.81	3 (2.9)	0.993
	Poorly	53 (52.0)	51 (50)	***0.017***	9 (8.82)	0.495	12 (11.8)	0.993
K-i67	<50%	15 (14.7)	11 (10.8)	***0.043***	5 (4.9)	***0.042***	3 (2.9)	0.921
	≥50%	87 (85.3)	80(78.4)		14 (13.7)		13 (12.7)	
P16	Negative	42 (41.2)	38 (37.3)	0.732	13 (12.7)	***0.011***	9 (8.8)	0.143
	Positive	60 (58.8)	53 (52.0)		6 (5.9)		7 (6.9)	
Survival status	Alive	77 (75.5)	69 (67.6)	0.822	13 (12.7)	0.399	11 (10.8)	0.054
	Dead	25 (24.5)	22 (21.6)		6 (5.9)		5 (4.9)	

The age of onset ranged from 42 to 80 years. Smoking index was calculated in Pack-Years (PY), with 20 cigarettes usually equating to 1 pack, and PY = number of cigarettes per day/20 * years of smoking. Based on the PY, the group was divided at the median value of 10. According to the 8th AJCC TNM staging system, there were 59 cases (57.8%) in stages I/II, and 83 cases (81.4%) in stages III/IV.

### Univariate/Multivariate logistic analysis

Univariate logistic analysis (Table 1) indicated that the anatomical subsite of OPSCC occurrence (*p* = 0.007), degree of differentiation (*p* = 0.017), and Ki-67 levels (*p* = 0.043) are risk factors affecting cervical lymph node metastasis in patients. Ki-67 levels ≥50% (*p* = 0.042) and positive P16 expression (*p* = 0.011) were associated with a reduced risk of multiple cervical lymph node metastases.

Factors with *p* < 0.05 were included in the multivariate logistic regression analysis for lymph node metastasis, indicating that the anatomical subsite of OPSCC occurrence (*p* = 0.034) is an independent risk factor affecting lymph node metastasis.

**Fig. 1:**
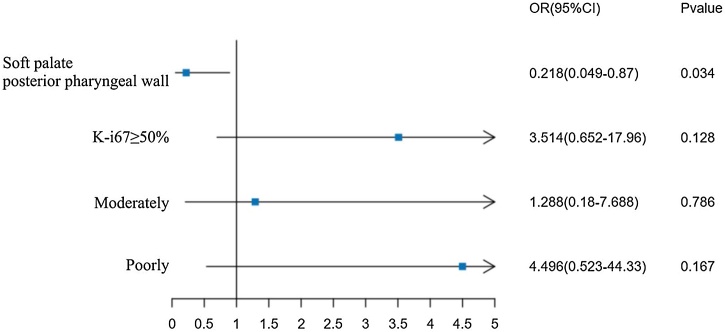
Forest plot of multivariate Logistic regression analysis for lymph node metastasis. Factors with *p* < 0.05 were included in the multivariate logistic regression analysis for lymph node metastasis, indicating that the anatomical subsite of OPSCC occurrence (*p* = 0.034) is an independent risk factor affecting lymph node metastasis.

The multivariate Logistic regression analysis forest plot is as follows (Fig. 1).

**Fig. 2:**
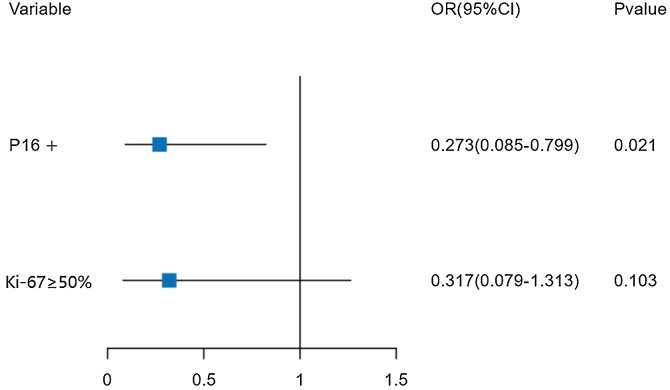
Forest plot of multivariate Logistic regression analysis for multiple lymph node metastases. Ki-67 ≥ 50% (*p* = 0.042) and P16 positivity (*p* = 0.011) were included in the multivariate logistic regression analysis of factors affecting multiple lymph node metastases. The results indicated that P16 status is an independent risk factor for multiple cervical lymph node metastases.

The multivariate Logistic regression analysis for multiple lymph node metastases indicates that P16 status is an independent risk factor affecting the occurrence of multiple lymph node metastases. The forest plot for the multivariate Logistic regression analysis is shown below (Fig. 2).

### Survival analysis

The impact of lymph node metastasis status and P16 status on the prognosis of patients with OPSCC.

**Fig. 3:**
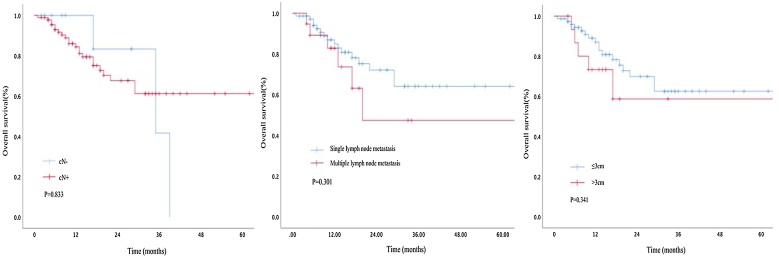
Kaplan–Meier survival analysis indicated that the imaging-assessed lymph node status (lymph node positive or negative, single or multiple, and metastatic lymph node diameter ≤3 cm or >3 cm) had no significant impact on prognosis.

**Fig. 4:**
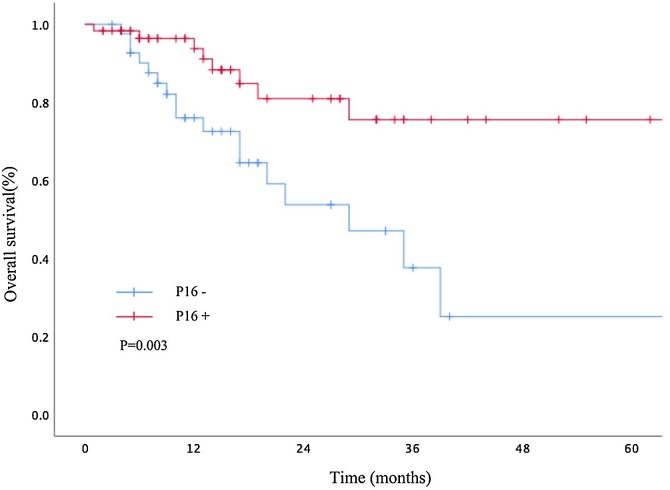
Kaplan-Meier survival analysis indicated that P16 status is a prognostic risk factor.

The follow-up period for the 102 patients ranged from 1 to 223 months, with an average follow-up of 20.4 months and a median follow-up of 14.4 months. Kaplan-Meier survival analysis indicated that lymph node metastasis status had no significant impact on prognosis. P16 status was identified as a risk factor affecting the prognosis (Figs. 3 and 4).

## Discussion

Over the past few decades, the incidence of HPV-OPSCC has been on the rise, but there are significant differences between countries [5], [6], [7], [8], [9]. Amanda F. Carlander and others [10] conducted a retrospective analysis of 49,564 OPSCC patients from 26 different countries between 2016 and 2021, finding that Lebanon and Sweden had HPV positivity rates of 85% and 70%, respectively. In the United States, the proportion of HPV-positive OPSCC patients increased from 54% in 2010 to 75% in 2015 [11]. Chinese scholars have also studied the HPV infection status of OPSCC patients in various regions, with the HPV positivity rate being relatively low (11%–21.28%) over the past decade [12], [13]. Professor Lu Xueguan's team [14] at Fudan University Cancer Hospital analyzed data from OPSCC patients between 2019 and 2022, finding that nearly 60% of all OPSCC patients were HPV-positive. Although HPV-positive status is associated with better chemoradiotherapy response and favorable prognosis, the steadily increasing incidence remains a cause for concern.

In Oral Squamous Cell Carcinoma (OSCC), the depth of invasion of the primary tumor is closely related to local recurrence and metastasis, and it is regarded as an important indicator for predicting lymph node metastasis and clinical prognosis [15]. In the eighth edition of the AJCC TNM staging for OSCC, the depth of invasion was included as a criterion for T staging. In contrast to OSCC, the new TNM staging for OPSCC still primarily bases the T stage on the maximum diameter of the primary lesion, without considering depth of invasion. However, HPV-OPSCC exhibits unique biological characteristics, with early lymph node metastasis often occurring even in smaller primary tumors.

It is well recognized in Head and Neck Squamous Cell Carcinoma (HNSCC) that lymph node metastasis is a known poor prognostic factor [16], often associated with lower survival rates and quality of life. Research by Lavaf A and others [17] confirmed that the presence of lymph node metastasis in HNSCC signifies lower disease-free survival and a worse prognosis, necessitating more aggressive treatment. In our retrospective analysis, the number of patients clinically assessed as having cervical lymph node metastasis accounted for 89.2%, which is higher than the 71.1% reported by previous researchers [18]. This may be due to the relatively underdeveloped economy in Shanxi Province, where public awareness of health is low. Many individuals often ignore or tolerate early discomfort, delaying medical consultation. As a result, many patients are diagnosed with a high rate of lymph node metastasis, and their clinical stage is more advanced, with stage III/IV accounting for 81.4% of cases. However, suspiciously positive lymph nodes did not affect the prognosis of OPSCC patients (*p* = 0.83). This conclusion is consistent with the clinical staging perspective of the 8th AJCC, which acknowledges that HPV-OPSCC exhibits different biological behavior and prognostic characteristics, suggesting that it should be staged as a distinct HNSCC entity. For OPSCC, both clinical and pathological staging should consider HPV status.

Immunohistochemistry (IHC) of P16 protein is considered the simplest and most practical method for detecting HPV infection [19], and various guidelines for the diagnosis and treatment of head and neck tumors also recommend P16 IHC testing to determine the association with HPV infection in OPSCC. As the incidence of HPV-OPSCC in China has been rising annually, the positive detection rate of P16 protein in OPSCC has also gradually increased, with our study showing that 58.8% of patients were P16 positive, almost approaching the rates in Western countries. Multiple studies have confirmed that HPV-OPSCC has a better prognosis compared to non-HPV-OPSCC. In our study, P16 status was an independent risk factor affecting patient prognosis (p = 0.038), with 1-year and 3-year survival rates for P16 positive patients being higher (93.8%, 75.5%) than those for P16 negative patients (72.5%, 37.6%). P16 status did not affect whether lymph nodes were metastatic (p = 0.732) or the size of the lymph nodes (p = 0.143), but it was a protective factor against multiple lymph node metastases, with P16 positive patients less likely to have multiple lymph node metastases (p = 0.021). Research by Kao. J and others [20] analyzed the impact of the number of metastatic lymph nodes on the prognosis of HNSCC patients, finding that a single lymph node metastasis resulted in a 5 year survival rate of 50% for patients, while multiple lymph node metastases reduced the survival rate to 33%, indicating a risk factor for prognosis. However, our study showed that multiple lymph node metastases (p = 0.301) did not have a statistically significant impact on patient prognosis. In the 8th AJCC, multiple ipsilateral lymph node metastases are classified as cN2b in P16 negative OPSCC, but as cN1 in P16 positive patients, thus diluting the impact of the number of ipsilateral metastatic lymph nodes on the prognosis of HPV-OPSCC patients. Previous scholars have reported that HPV-OPSCC patients are less likely to experience distant metastases. Our study found that patients with multiple lymph node metastases were more likely to develop distant metastases (*p* = 0.009), suggesting that P16 positivity may reduce the risk of multiple lymph node metastases, thereby decreasing the incidence of distant metastatic events, but this hypothesis requires further research for confirmation.

The lymph node diameter greater than 3 cm has been traditionally considered a risk factor affecting the prognosis in HNSCC. Over the years, the TNM staging guidelines for head and neck tumors have consistently used 3 cm as a demarcation line for different N stages. However, our study found that a lymph node diameter greater than 3 cm (*p* = 0.301) did not significantly impact patient prognosis. This might be due to the unique etiological factors and biological characteristics of HPV-OPSCC, leading to the 8th AJCC staging guidelines no longer using a lymph node diameter of more than 3 cm as a boundary for N stages in HPV-OPSCC.

OPSCC can occur in several subsites, including the palatine tonsils, base of the tongue, posterior pharyngeal wall, and soft palate. The anatomical location and lymphatic drainage patterns of these sites can affect the mode of cancer cell metastasis, including the regions to which they spread and the speed and severity of the spread. The palatine tonsils and the base of the tongue, due to their unique anatomical structures, often exhibit early lymph node metastasis, and the metastasized lymph nodes may be larger [21]. Our study found that OPSCC originating from the palatine tonsils and base of the tongue was more likely to metastasize to cervical lymph nodes (*p* =  0.007) and was an independent risk factor for lymph node metastasis. Among 36 patients who presented with enlarged cervical lymph nodes as the initial symptom, 31 (86.1%) had cancer of the palatine tonsils or base of the tongue. Although the sub-anatomical location did not significantly affect patient survival time, the treatment strategy for cancer of the palatine tonsils and base of the tongue usually requires consideration of extensive cervical lymph node dissection or radiotherapy. In cases where lymph node metastasis occurs early in the head and neck region and the primary site is unknown, the palatine tonsils or base of the tongue should be considered as possible primary sites for investigation [22].

Larsen et al. [23] confirmed through research that the pathological grading of oral squamous cell carcinoma is correlated with tumor recurrence and cervical lymph node metastasis. Our study found that poorly differentiated squamous cell carcinoma accounted for the highest proportion (53.9%) among OPSCC patients, and pathological grading is a risk factor for predicting cervical lymph node metastasis (p = 0.017). The risk of cervical lymph node metastasis in poorly differentiated squamous cell carcinoma is 11 times that of well-differentiated squamous cell carcinoma (OR = 10.929, 95% CI 1.546–77.264). However, unlike other HNSCCs, the impact of pathological grading on the prognosis of OPSCC is limited [24] (*p* = 0.492), which is also associated with the presence of HPV-positive status [25].

Ki-67 is a nuclear protein expressed during cell proliferation and is often used as an indicator of tumor proliferative activity. Some researchers have confirmed that high expression of Ki-67 is an independent marker affecting the prognosis of patients with oral squamous cell carcinoma [26]. In our study, Ki-67 ≥ 50% did not have a statistically significant impact on the prognosis of OPSCC patients. Patients with Ki-67 ≥ 50% were more likely to experience lymph node metastasis but were protected against multiple lymph node metastases. We consider that although high expression of Ki-67 is a poor prognostic factor for many malignant tumors, the adverse prognosis brought by high expression of Ki-67 may be mitigated in an HPV-positive environment for OPSCC, and a higher Ki-67 index needs to be combined with other related biomarkers to be more meaningful.

There are many other biological markers that may influence the prognosis of OPSCC, such as P53, Programmed Death receptor-1 (PD-1), Programmed Death Ligand-1 (PD-L1), Epidermal Growth Factor Receptor (EGFR), and Tumor-Infiltrating Lymphocytes (TILs) [27]. Combining these potential prognostic markers with traditional factors can enhance the ability to stratify risk in OPSCC patients. We will continue to explore these areas in future studies.

## Conclusion

In summary, despite the retrospective nature of our study, the small number of cases, and the short follow-up period, which are limitations, it is still apparent that the biological characteristics of HPV-OPSCC differ due to regional, economic, and cultural differences. HPV-OPSCC has distinct cervical lymph node metastasis characteristics compared to other HNSCCs, with its status of cervical lymph node metastasis not significantly impacting patient prognosis. The increase in the incidence of HPV-OPSCC first occurred in developed regions of Europe and America, and with economic development, the proportion of HPV-positive OPSCC in Shanxi, a less developed region of China, has also surpassed that of oropharyngeal cancer caused by smoking and alcohol. Given the unique biological and clinical characteristics of HPV-OPSCC, accurate prognostic risk stratification of OPSCC patients by incorporating specific biological markers, and exploring individualized comprehensive treatments, should be a key direction in the current field of head and neck cancer treatment.

## Ethical considerations

The collected information and data are obtained through legal means, from hospital medical records, with permission and authorization, and will not violate any ethical guidelines.

## Funding

None.

## Data availability statement

The data presented in this study are available on request from the corresponding author after approval of a data use proposal. The data are not publicly available due to privacy and ethical restrictions.

## Declaration of competing interest

Report potential conflicts of interest that are not upcoming or existed in the past 24 months.

## References

[1] Sung H., Ferlay J., Siegel R.L., Laversanne M., Soerjomataram I., Jemal A. (2021). Global cancer statistics 2020: GLOBOCAN estimates of incidence and mortality worldwide for 36 cancers in 185 countries. CA Cancer J Clin..

[2] Chaturvedi A.K., Engels E.A., Pfeiffer R.M., Hernandez B.Y., Xiao W., Kim E. (2023). Human papillomavirus and rising oropharyngeal cancer incidence in the United States. J Clin Oncol..

[3] Gillison M.L., D’Souza G., Westra W., Sugar E., Xiao W., Begum S. (2008). Distinct risk factor profiles for human papillomavirus type 16-positive and human papillomavirus type 16-negative head and neck cancers. J Natl Cancer Inst..

[4] Lydiatt W.M., Patel S.G., O’Sullivan B., Brandwein M.S., Ridge J.A., Migliacci J.C. (2017). Head and Neck cancers-major changes in the American Joint Committee on cancer eighth edition cancer staging manual. CA Cancer J Clin.

[5] Zamani M., Grønhøj C., Jensen D.H., Carlander A.F., Agander T., Kiss K. (2020). The current epidemic of HPV-associated oropharyngeal cancer: an 18-year Danish population-based study with 2,169 patients. Eur J Cancer..

[6] Argirion I., Zarins K.R., McHugh J., Cantley R.L., Teeramatwanich W., Laohasiriwong S. (2020). Increasing prevalence of HPV in oropharyngeal carcinoma suggests adaptation of p16 screening in Southeast Asia. J Clin Virol..

[7] Kim Y., Joo Y.H., Kim M.S., Lee Y.S. (2020). Prevalence of high-risk human papillomavirus and its genotype distribution in head and neck squamous cell carcinomas. J Pathol Transl Med..

[8] Donà M.G., Rollo F., Pich B., Spriano G., Moretto S., Covello R. (2020). Evolving profile of hpv-driven oropharyngeal squamous cell carcinoma in a national cancer institute in italy: a 10-year retrospective study. Microorganisms..

[9] Mena M., Frias-Gomez J., Taberna M., Quirós B., Marquez S., Clavero O. (2020). Epidemiology of human papillomavirus-related oropharyngeal cancer in a classically low-burden region of southern Europe. Sci Rep..

[10] Carlander A.F., Jakobsen K.K., Bendtsen S.K., Garset-Zamani M., Lynggaard C.D., Jensen J.S. (2021). A contemporary systematic review on repartition of HPV-positivity in oropharyngeal cancer worldwide. Viruses..

[11] Faraji F., Rettig E.M., Tsai H.-L., El Asmar M., Fung N., Eisele D.W. (2019). The prevalence of human papillomavirus in oropharyngeal cancer is increasing regardless of sex or race, and the influence of sex and race on survival is modified by human papillomavirus tumor status. Cancer..

[12] Meng H.-X., Miao S.-S., Chen K., Li H.-N., Yao G., Geng J. (2018). Association of p16 as prognostic factors for oropharyngeal cancer: evaluation of p16 in 1470 patients for a 16-year study in Northeast China. Biomed Res Int..

[13] Xu S., Sun B., Zhou R., Shi C., Han Y., Li J. (2020). Evaluation of p16 as a surrogate marker for transcriptionally active human papillomavirus status of oropharyngeal squamous cell carcinoma in an eastern Chinese population. Oral Surg Oral Med Oral Pathol Oral Radiol..

[14] Wei Y., Xu T., Li C. (2023). CD161 characterizes an inflamed subset of cytotoxic T lymphocytes associated with prolonged survival in human papillomavirus driven oropharyngeal cancer. Cancer Immunol Res..

[15] Anderson E.M., Luu M., Chung E.M., Gay C., Clair J.M.-S., Ho A.S. (2023). Re-examining predictors of pathologic lymph node positivity in clinically node negative oral cavity cancer. Oral Oncol..

[16] Matija M., Ivica L. (2021). Lymph node characteristics and their prognostic significance in oral squamous cell carcinoma. Head Neck..

[17] Lavaf A., Genden E.M., Cesaretti J.A., Packer S., Kao J. (2008). Adjuvant radiotherapy improves overall survival for patients with lymph node-positive head and neck squamous cell carcinoma. Cancer..

[18] Bauwens L., Baltres A., Fian D.-J., Zrounba P., Buiret G., Fleury B. (2021). Prevalence and distribution of cervical lymph node metastases in HPV-positive and HPV-negative oropharyngeal squamous cell carcinoma. Radiother Oncol..

[19] Augustin J.G., Lepine C., Morini A., Brunet A., Veyer D., Brochard C. (2020). HPV detection in head and neck squamous cell carcinomas: what is the issue?. Front Oncol..

[20] Kao J., Lavaf A., Teng M.S., Huang D., Genden E.M. (2008). Adjuvant radiotherapy and survival for patients with node-positive head and neck cancer: An analysis by primary site and nodal stage. Int J Radiat Oncol Biol Phys..

[21] Stjernstrøm K.D., Jensen J.S., Jakobsen K.K., Grønhøj C., von Buchwald C. (2019). Current status of human papillomavirus positivity in oropharyngeal squamous cell carcinoma in Europe: a system-artic review. Acta Otolaryngol..

[22] Editorial Committee of Head and Neck Surgery Group, Chinese Society of Otorhinolaryngology Head and Neck Surgery, Chinese Medical Association (2024). Expert consensus on diagnosis and treatment of cervical metastatic squamous cell carcinoma of unknown primary (2024). Chin J Otorhinolaryngol Head Neck Surg.

[23] Larsen S.R., Johansen J., Sørensen J.A., Krogdahl A. (2009). The prognostic significance of histological features in oral squamous cell carcinoma. J Oral Pathol Med..

[24] Mahal B.A., Catalano P.J., Haddad R.I., Hanna G.J., Kass J.I., Schoenfeld J.D. (2019). Incidence and demographic burden of HPV-associated oropharyngeal head and neck cancers in the United States. Cancer Epidemiol Biomarkers Prev..

[25] Lewis J.S. (2017). Morphologic diversity in human papillomavirus-related oropharyngeal squamous cell carcinoma: catch me if you can!. Mod Pathol..

[26] Jing Y., Zhou Q., Zhu H., Zhang Y., Song Y., Zhang X. (2019). Ki-67 is an independent prognostic marker for the recurrence and relapse of oral squamous cell carcinoma. Oncol Lett..

[27] Wongpattaraworakul W., Choi A., Buchakjian M.R., Lanzel E.A., Rajan Kd A., Simons A.L. (2024). Prognostic role of tumor-infiltrating lymphocytes in oral squamous cell carcinoma. BMC Cancer..

